# Effect of S-methyl-l-thiocitrulline dihydrochloride on rat micturition reflex

**DOI:** 10.1590/S1677-5538.IBJU.2015.0153

**Published:** 2016

**Authors:** Jeová Nina Rocha

**Affiliations:** 1 Divisão de Urologia, Faculdade de Medicina Ribeirão Preto, Universidade de São Paulo, SP, Brasil

**Keywords:** Urethra, Urinary Sphincter, Artificial, Infusions, Intraventricular, Nitric Oxide Synthase

## Abstract

**Objective:**

To evaluate the effect of neuronal nitric oxide synthase on the striated urethral sphincter and the urinary bladder.

**Materials and Methods:**

A coaxial catheter was implanted in the proximal urethra and another one in the bladder of female rats, which were anesthetized with subcutaneous injection of urethane. The urethral pressure with saline continuous infusion and bladder isovolumetric pressure were simultaneously recorded. Two groups of rats were formed. In group I, an intrathecal catheter was implanted on the day of the experiment at the L6-S1 level of the spinal cord; in group II, an intracerebroventricular cannula was placed 5-6 days before the experiment.

**Results:**

It was verified that the group treated with S-methyl-L-thio-citrulline, via intrathecal pathway, showed complete or partial inhibition of the urethral sphincter relaxation and total inhibition of the micturition reflexes. The urethral sphincter and the detrusor functions were recovered after L-Arginine administration. When S-methyl-L-thio-citrulline was administered via intracerebroventricular injection, there was a significant increase of urethral sphincter tonus while preserving the sphincter relaxation and the detrusor contractions, at similar levels as before the use of the drugs. Nevertheless there was normalization of the urethral tonus when L-Arginine was applied.

**Conclusions:**

The results indicate that, in female rats anaesthetized with urethane, the nNOS inhibitor administrated through the intrathecal route inhibits urethral sphincter relaxation, while intracerebroventricular injection increases the sphincter tonus, without changing bladder function. These changes were reverted by L-Arginine administration. These findings suggest that the urethral sphincter and detrusor muscle function is modulated by nitric oxide.

## INTRODUCTION

The lower urinary tract function, including bladder, and striated urethral sphincter, fills/ stores and empties/voids urine in two distinct phases. The gradual filling/storage of urine by the bladder (under low pressure) involves a progressive increase of the urethral sphincter muscle tonus, while simultaneously maintaining the urethra closed. The integrity and coordination between the autonomic and somatic nervous systems is important in order to preserve continence and allow micturition. Emptying/voiding requires rapid pressure elevation and concomitant relaxation of the urethral sphincter (cross-talk mechanism), facilitating urine drainage (1, 2).

Bladder function has been studied using electric stimulation as well as by drug administration. Electric or chemical stimulation of the dorsolateral pontine tegmentum, also described as the pontine micturition center (PMC), provokes micturition due to simultaneous bladder contraction and urethral sphincter motoneuron inhibition, while microinjections with inhibitory drugs in the PMC block the micturition reflex (3). Electric stimulation of the nucleus paracoelureus causes contraction of the pelvic floor muscle and, as consequence, increases urethral pressure, whereas injury to the nuclei of this area evokes detrusor hypertonia and urethral relaxation (4). For this relaxation and contraction mechanism of the detrusor and urethral sphincter to happen, it has been proposed that descendent fibers from the PMC project directly onto the preganglionic parasympathetic nuclei, on the dorsal commissure nuclei and on the intermediumlateralis column nuclei (4). The activation of these preganglionic nuclei stimulates pelvic ganglia and inhibits interneurons that have projections in Onuf's nuclei. These interneurons release two important neurotransmitters: γ-aminoglutamic acid (GABA) and glycine (5–8) that facilitate the urethral sphincter relaxation. Nevertheless, this relaxation is not observed when strychnine, a glycine inhibitor, is administrated before any external stimulation. This strongly suggests the possibility of the involvement of these neurotransmitters in the urethral activity (7, 9, 10). Furthermore, Buss & Shefchyk verified that electric stimulation applied directly on the preganglionic parasympathetic nuclei leads to relaxation of the striated urethral sphincter with consequent reduction of intraurethral pressure (6).

The micturition and storage complex mechanism has the participation of several neurotransmitters, including nitric oxide (NO) at the peripheral and central nervous system (11). There are reports of high densities of nitric oxide synthase (NOS), an enzyme that catalyzes the amino acid L-Arginine [L-ARG] to convert to L-citrulline and NO in the presence of oxygen and nicotinamide adenine dinucleotide phosphate (NADPH), in the spinal cord dorsal horn, especially in the superficial area, in the dorsal commissure and in the site of the preganglionic sympathetic and parasympathetic nuclei (12–14). Therefore, NO induces striated urethral sphincter (SUS) in vitro relaxation (13) and when reflexly induced (15–17).

It has been proposed that NO acts as an important mediator in non-adrenergic non-cholinergic neurotransmitters (NANC) in the smooth and striated muscles of the lower urinary tract, since NO synthase (NOS) is present in micturition reflex neuronal fibers. These include primary sensitive neurons, spinal interneurons, preganglionic sympathetic and parasympathetic neurons in the spinal cord, postganglionic parasympathetic neurons in the peripheral ganglia, in lower urinary tract motoneurons and in afferent fiber dorsal root ganglia (15, 18, 19).

As it is not well established what type of NOS isoform is involved in the bladder outlet function, the aim of this work was to study the effect of the neuronal NOS (nNOS) isoform on the detrusor and SUS during reflexogenic contractions in normal female rats.

## MATERIAL AND METHODS

### Ethical approval

The protocol applied in this experiment was approved by the Committee of Ethics of the Ribeirão Preto Medical School, University of S. Paulo. All efforts were undertaken to minimize the suffering and reduce the number of animals used in this experimental protocol.

### Surgical Procedure

Female Wistar rats, weighting 230-270g (45-50 day old), were anesthetized with urethane (1.0g/kg, subcutaneously). After a lower abdominal incision (~1cm), the bladder was exposed and a polyethylene catheter (PE-50; id=0.58mm; od: 0.96mm; Clay Adams, Parsipanny, NJ, USA), with the end flared by heat to create a small collar, was implanted through the bladder's anterior wall. A coaxial (double-lumen made in our laboratory) catheter consisting of an outer tube (PE-160; od=1.57mm; id=1.14mm; Clay-Adams) and an inner tube (PE-50) was inserted into the proximal urethra through the bladder dome, separating the bladder and urethra, with no risk of neural damage to the bladder neck. Both catheters were separately tied with 4-0 silk, and attached to the pressure transducers (World Precision Instruments) to record urethra and urinary bladder pressures. For isovolumetric recording of the bladder pressure, the ureters were transected at the level of the aortic bifurcation so that diuresis did not change bladder volume during the experiment. The distal ends of the ureters were tied, and a PE-10 catheter (id=0.28mm; od=0.61mm; Clay-Adams) was placed in the proximal section of each ureter for spontaneously draining urine out of the abdominal cavity. All data were collected and stored in the computer by Windaq software (DATAQ Instruments Inc., Akron, OH, USA). At the end of the experiments, the animals were euthanatized by exsanguination. Following this procedure, a dye was injected in the subarachnoid space and in the lateral ventricle, and a lumbar laminectomy or craniotomy was performed in every rat to confirm the catheter or cannula position, respectively.

### Cystourethrometry

**Figure 1:**
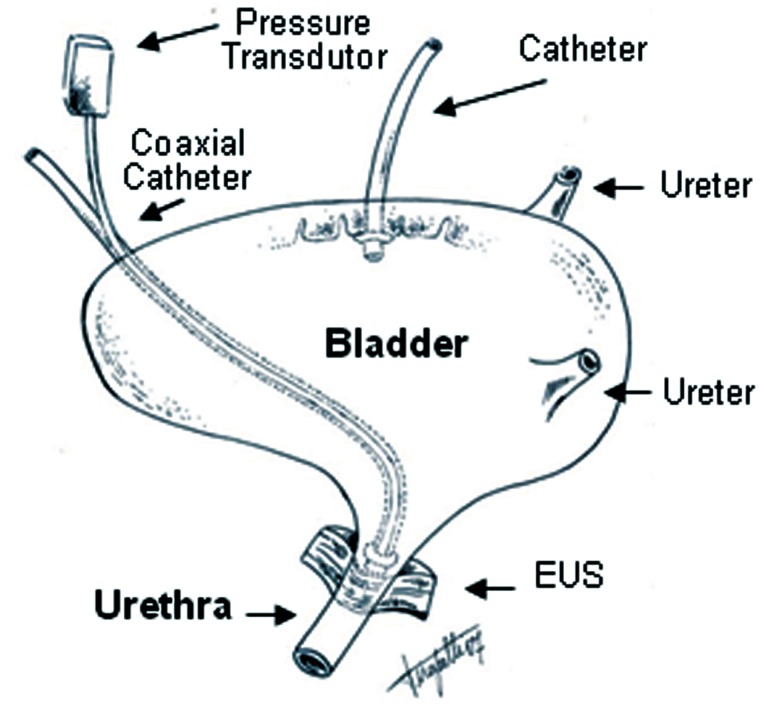
Representative diagram of experimental model applied in this study, and simultaneous evaluation of the urinary bladder isovolumetric pressure and isotonic urethral pressure of normal female rats, anesthetized with urethane.

Using a micro-pump (Kd Scientific Infusion Pump, Model 780.200, Ser. #201755, USA), a warm saline (37°C, 0.9%) infusion was injected through the PE-50 catheter, at a flow rate of 0.02mL/min until the bladder contraction attained a pressure higher than 15cmH_2_O, at which point the infusion was interrupted. The urethra was continually infused (with the micro-pump) with saline solution through the coaxial catheter at a rate of 0.075mL/min to record the urethral perfusion pressure (UPP), and the vesical pressure under isovolumetric conditions (Figure-1). The saline infused into the urethra was allowed to drain freely through the urethral meatus.

### Implantation of the Intrathecal (i.t.) Catheter

The i.t. implantation of a catheter (PE-10) in the rats (n=7) was performed on the same day of the experimental procedure. A special needle was placed in the vertebral canal through the L_5_-L_6_ intervertebral space. The correct positioning of this needle in the subarachnoid space was confirmed by the tail-flick reflex of the rat. Following this procedure, a PE-10 catheter containing 10% heparin was passed through the needle, progressing 2.0cm into the intravertebral canal, and the distal tip of this catheter was placed exactly between segments L_6_-S_1_ of the spinal cord. The needle was then removed, and the catheter was tied to the subcutaneous tissue with 4-0 silk.

### Implantation of the Intracerebroventricular (i.c.v) Cannula

Rats (n=6) were anesthetized with ketamine (75mg/kg) and xilazine (7.5mg/kg), both administered intramuscularly. The animals were placed in a stereotaxic frame (David Kopf Instrument, USA) in a dorsal recumbent position. A small burr hole, 1mm in diameter, was made in the parietal cortex using a dental drill, care being taken to avoid damage to the dura during drilling. A 23^G^ cannula, 10.5mm in length (made in our laboratory) was implanted in the right lateral ventricle (1.0mm posterior to the bregma, 1.6mm lateral to the midline, and 3.4mm below the surface of the skull), according to Paxino's and Watson's coordinates (20). The cannula was fixed to the skull with autopolimerizant acrylic cement. A metallic mandrill was placed in this cannula to close the system, and fixated with the same acrylic cement. G-Procaine Penicillin (8.000UI, intramuscular) was administered immediately after surgery.

The rats were housed in cages with wood shavings, at a controlled temperature (22±0.2°C), with a light/dark cycle of 12/12h, and had free access to food and water. Experimental recordings were performed 5-6 days after cannula implantation.

To evaluate the involvement of NO in the function of the rat's detrusor and striated urethral sphincter (under normal conditions) we administrated, through either i.t. or i.c.v. spaces the following drugs: (a) S-methyl-L-thio-citrulline dihydrochloride (L-SMTC), a selective potent inhibitor of the NOS isoform enzyme, at a single dose of 1.0μmol (mixed with saline 7μL), followed by flushing with 10μL of artificial cerebrospinal fluid (aCSF) (composition in g/L: NaCl=8.1; KCl=0.25; Na_2_HPO_4_=0.08; MgCl_2_6H_2_0=0.1; NaHC0_3_=1.72; CaCl_2_2H_2_O=0.14; (NH_2_)2CO=1.3; Glucose=0.6, pH=7.4, gassed previously with 95% O_2_/5% CO_2_) and (b) L-Arginine, a physiological substrate for NOS and the precursor of NO, at single dose of 10μmoL (10μL), with an additional 10μL aCSF for flushing. The drug administration was performed slowly (5μL/ min), using a Hamilton micro-syringe (10μL). Catheter volume was approximately 7μL.

### Drugs

The drugs used in this experiment were: S-methyl-L-thio-citrulline dihydrochloride (Calbiochem Inc, USA); L-Arginine and Urethane (Sigma Chemical Co. USA); Xilazine Chloride (Carbier SA, Spain); Ketamine Chloride (Cristália SA, Brazil) and G Procaine Penicillin (Euro-farma Laboratórios, Brazil).

### Statistical analysis

The results were expressed by mean±the standard error of the mean. The ANOVA test was used for statistical analysis, followed by post hoc Bonferroni's multiple-comparisons test (multiple-group comparison). The values were considered statistically significant when P<0.05. Graphs were generated using GraphPad 5.0 Prism Program (GraphPad Software, Inc., San Diego, CA, USA).

## RESULTS

### Cystourethrometry

**Figure 2:**
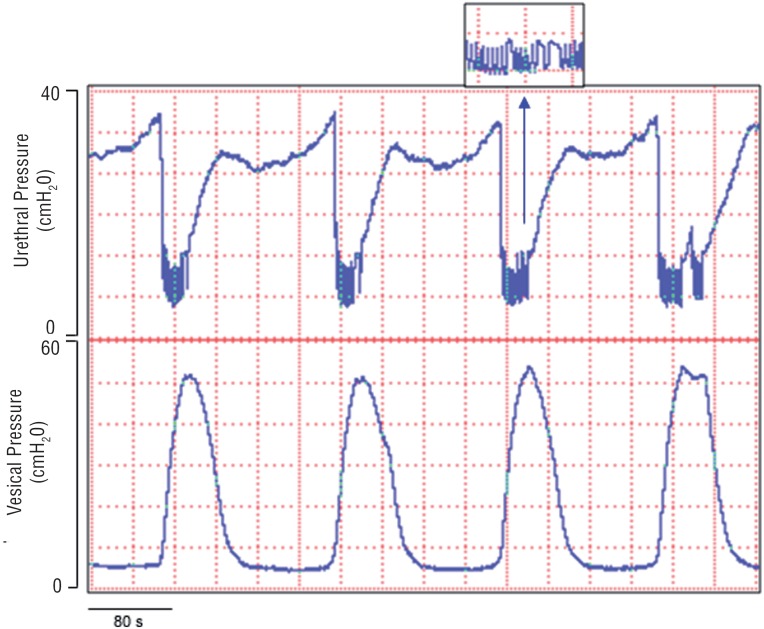
Simultaneous and independent recording of both the vesical isovolumetric and urethral pressure in a normal female rat anesthetized with urethane. During the maximal relaxation of the urethral pressure high frequency oscillations occur (amplified square), and they disappear when the bladder pressure decreases and the urethral pressure returns to its baseline.

**Figure 3:**
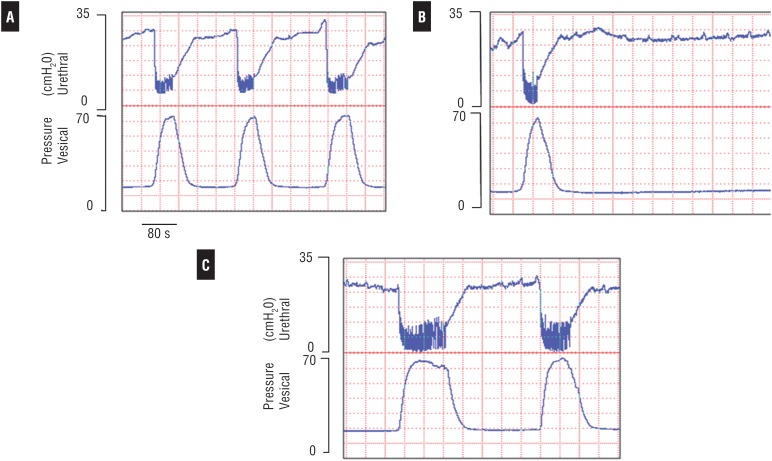
(GI) – Simultaneous and independent recordings of the bladder isovolumetric and urethral sphincter pressure of a female rat. Rat anesthetized with urethane in the control phase (A), after administration of L-SMTC (B) and L-Arginine (C). The administration of nNOS (1 μmol, i.t.), a selective inhibitor, and isovolumetric contractions of the detrusor and the relaxation of the urethral sphincter were abolished completely. These functions were restored after the L-ARG (10 μmol, i.t.), a NO precursor, albeit the detrusor contractions were prolonged. This record also shows bursting (high frequency oscillations) during the maximal urethral relaxation and the contraction of the detrusor (A, C). This bursting was significant during the detrusor contraction maximal spike and ended at the return to the baseline of bladder pressure.

**Figure 4:**
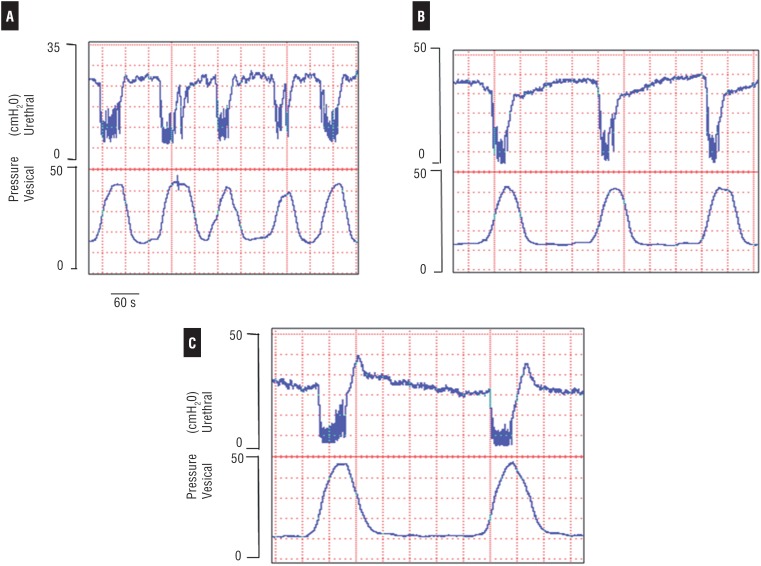
(GII) - Simultaneous and independent records of the bladder isovolumetric and urethral sphincter pressure of a female rat. Data was collected in the rat anesthetized with urethane in the control phase (A), after L-SMTC (B) and L-ARG (C) administration. The administration of the nNOS (1 μmol, i.c.v.) did not abolish isovolumetric contractions of the detrusor and the relaxation of the urethral sphincter, but increased the interval and discreet duration of the detrusor contractions. These changes were not restored after the administration of L-ARG, however, the sphincter tonus was reduced and the miccional interval was elongated. This recording also shows that there were no changes in bursting (high frequency oscillations) during the maximal urethral relaxation and in maximal detrusor contractions (A, B, C).

During the maximal isovolumetric contraction of the detrusor a nadir urethral pressure occurred, simultaneously exhibiting a coordinated reciprocity between the bladder contraction and sphincter relaxation at regular intervals (Figure-2). It was also verified that in all animals the decrease of urethral pressure preceded, by a short period, the increase of intravesical pressure. The maximal amplitude of the detrusor contraction coincided with maximal relaxation of the urethral pressure. Bursting was observed in all records, and these reflexes only occurred during the maximal amplitude of the bladder contractions (Figures 2, 3A-C, and 4A-C). These oscillations were suppressed immediately after restoration of urethral pressure to the baseline, and simultaneous reduction of the detrusor contraction (Figure-2).

### Intrathecal (i.t.) Drug

**Figure 5:**
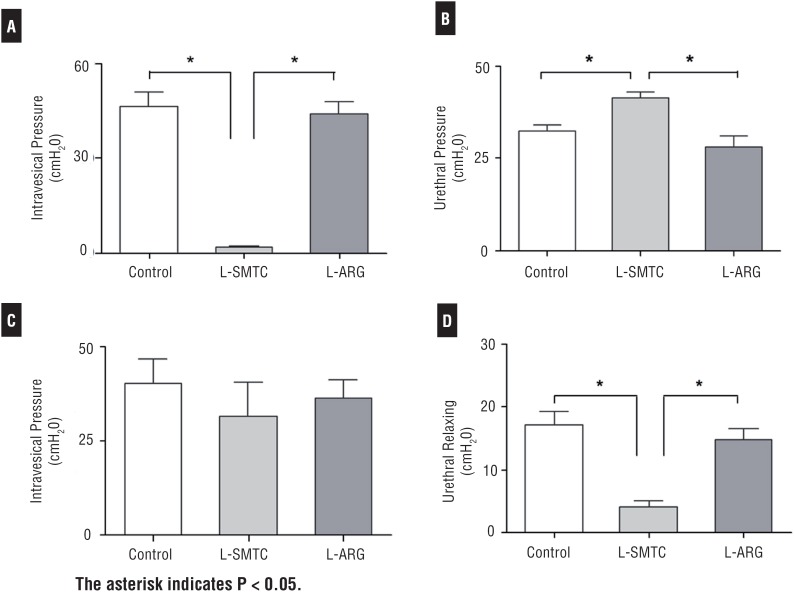
Effect of the administration of i.t. (A, B) and i.c.v. (C, D) L-SMTC on intravesical pressure and on urethral sphincter relaxation during the detrusor isovolumetric contraction of female rats anesthetized with urethane. With drug administration (L-SMTC, 1 μmoL, i.t.) an expressive inhibition of the detrusor contraction (A), and urethral sphincter nadir relaxation inhibition (B) occurred. After L-ARG (10 μmol, i.t.) administration bladder and urethral function were restored (A, B). Nevertheless, when the injection was performed via i.c.v. there were no significant changes in the intravesical pressure (C), but a statistically significant inhibition of the urethral tonus that was reversed after the L-ARG administration (D).

Five to ten minutes after L-SMTC administration (1μmol, i.t.) total reduction of the detrusor contraction was observed as well as significant suppression of the urethral sphincter relaxation (Figures 3A and B, Table-1). After L-ARG administration (10μmol, i.t.) the vesicourethral function was restored. Results showed maximum amplitude of the bladder contractions, and relaxation of the urethral sphincter, which were similar to the recordings before administration of the L-SMTC drug. After restoration of the vesicourethral function, we noted that the tonus of the urethral sphincter was slightly increased. Following the administration of the L-ARG, there were no significant changes in the magnitude of the values of bladder pressure, but there was an increase in the duration and interval of the detrusor isovolumetric contractions. At the same time, there were no changes to the magnitude of the intravesical pressure (Figures 3C, 5A and B, Table-1).

**Table 1:** Cistouretromanometry in female Wistar rats after administration of L-SMTC and L-ARG.

		Control	L-SMTC	L-ARG
i.t.	Vesical pressure (cmH_2_O)	46.4±4.8	1.4±0.2	44.5±3.7
	Urethral pressure (cmH_2_O)	32.5±1.7	41.3±1.7	28.2±2.7
i.c.v.	Vesical pressure (cmH_2_O)	40.2±3.2	31.5±9.2	36.5±4.8
	Urethral pressure (cmH_2_O)	-17.2±-1.3	-4.7±-1.3	-14.9±-1.7

**L-SMTC** = S-methyl-L-Tiocitrullina; **L-ARG** = L-Arginine **i.t.** = intrathecal; **i.c.v**. = intracerebroventricular

### Intracerebroventricular (i.c.v.) Drug

After L-SMTC administration (1μmoL i.c.v.), there were no significant changes to bladder pressure, but there was a slight increase in the duration of the detrusor contraction. All but one animal, showed an increase of the urethral tonus. At the same time changes in the urethral relaxation were not observed, maintaining a similar record to that noted before the L-SMTC administration (Figures 4B and C, 5C and D, Table-1). After L-ARG administration, mean values of the urethral relaxation amplitude were similar to those before the L-SMTC injection. There was a slight decrease in bladder pressure amplitude, but without statistical significance (Figure-5C).

## COMMENTS

In this study it was verified that changes to both bladder and striated urethral sphincter of normal rats occurred after using the L-SMTC and L-ARG drugs. The main observation after the administration of L-SMTC (1μmol, i.t.) was an inhibition of the detrusor contraction and total reduction of the urethral sphincter relaxation amplitude. We noticed that i.t. delivery maintained the pressure baseline, whereas i.c.v. injection led to a slight increase of the striated urethral sphincter pressure. After L-ARG administration (1μmol i.t. and i.c.v.), whole recovery of the bladder and urethral function was observed. It is quite probable that the effect that provoked the relaxation inhibition of the urethral sphincter was due to the inhibition of NO production, since the nitrergic neurotransmitter is directly involved in smooth muscle relaxation of the urethra of several animal species (15). This interpretation is also consistent with the observation of in vivo i.t. administration of N^G^-Nitro-L-Arginine (L-NA), an NOS inhibitor, which completely suppresses urethral relaxation (19).

In the present study, this effect was observed by using the nNOS inhibitor as it hindered the urethral sphincter relaxation (maintaining the pressure on the baseline) as well as on the detrusor depression, caused by drug administration directly in the spinal cord segment (L_6_-S_1_). Interestingly, the same injection, administrated in the supraspinal region, produced an increase of the sphincter tonus without modifying its function. This fact suggests that this enzyme has diverse physiologic actions in different intracerebroventricular sites.

The apparent discrepancy between the responses of the L-SMTC administrated via i.t. and i.c.v. has not been well elucidated. It is important to consider that NO is expressed in many nuclei of the PMC such as in the locus coeruleus alpha, periacquecdutal gray, and nucleus paragigantocellularis (21). Indeed, NO may have a modulatory function in the release of the different neurotransmitters involved in the micturition reflex. On the other hand, it has been ascribed to the nNOS enzyme the inhibition in preganglionic parasympathetic nuclei found in the spinal cord segment L_6_-S_1_ (22). In this case, it has been also suggested that NO released from efferent parasympathetic fibers in the lower urinary tract has an inhibitor effect on the afferent fibers (13); and NO has been identified as a neurotransmitter in the urethral sphincter relaxation induced during the micturition reflex (23).

It has been accepted that responses observed in this study, after the administration of L-SMTC and L-ARG, via i.c.v., are the result of the action of these drugs on the PMC or on paraventricular nuclei found adjacent to the ventricle wall. As the nNOS isoform concentration in the paraventricular region is significant (24), it is consistent to attribute that the increased tonus of the urethral sphincter, after the i.c.v. administration of L-SMTC, is due to the high concentration of NOS in the supraspinal central nervous system. Furthermore, the activation and restoration of the urethral sphincter function verified in the present experiment after the L-SMTC and L-ARG injections in the lateral ventricle is indicative that the PMC or paraventricular nuclei found adjacent to the ventricle wall are influenced by these drugs (24). This possibility has as basis after specific radioligand injections [^14^C]-L-NA-(N^G^-Nitro-Arginine), [^14^C]L-Citrulline and 7-Nitro-Indazole (L-NIO). These drugs were readily detected in the adjacent tissue of the ventricle wall, and their action spread quickly from the infusion site (25, 26).

In this case, it is reasonable to accept that responses observed in this study, after the administration of L-SMTC and L-ARG, via i.c.v., are a result of the action of these drugs on the PMC or on paraventricular nuclei found adjacent to the ventricle wall. Moreover, it is possible that the cerebrospinal fluid of the lateral ventricles drains the drugs to the third and fourth ventricles, and through the interventricular aqueduct, they flow to the periaqueductal nuclei, and to area close to the tegmentum pontinum dorsomedialis and to the PMC. In this context, one should consider that neurons in the ventrolateral region of the periaqueduct have projections in the Barrington's nuclei (25–27).

The full or partial suppression of the urethral relaxation amplitude, using nNOS enzyme selective inhibitor via i.t. administration is consistent with the results found by Pullen and Humphreys (27). They identified, through histochemical studies, a high density of nNOS, but not eNOS and iNOS isoforms, in the motor somatic nuclei of the lumbosacral segment (L_6_-S_1_), in the ventrolateral region, in the ventromedial region and in Onuf's nuclei. Furthermore, it has been proposed that cholinergic somatic innervations of the sphincter, via pudendal nerve, contain the non-adrenergic non-cholinergic (NANC) neurotransmitter NO that induces urethral sphincter relaxation (28). A study that supports this observation was performed in our laboratory using female rats. It was verified an increase of the urethral resistance when N^G^-Nitro-L-Arginine Methyl Ester (L-NAME) was i.t. or i.c.v. administered, and this resistance was significantly reduced after the injection of L-ARG (unpublished observations).

Another relevant comment was a study performed with knockout mice (targeted deletion of the nNOS gene). The authors noted that these animals showed dysfunctional urinary outlets and hypertrophied and dilated bladders (29, 30). Moreover, they observed that these bladder and sphincter muscles did not relax under electrical stimulation. Similar data were observed when rats were treated with aminoguanidine, a selective inhibitor of the iNOS isoform (31). These observations are indicative that the drug used in this study (L-SMTC) can be effective in a similar way, inhibiting the relaxation or increasing the urethral sphincter tonus mediated by NO and, in consequence, causing an increase of bladder capacity and compliance.

In summary, results of this research indicate that in female rats the effect of nNOS (L-SMTC) causes inhibition of the detrusor contraction and inhibition of the urethral sphincter relaxation following drug injection via i.t.; the urethral sphincter tonus was unaltered, without significant changes in its function or in reflexes of the detrusor when the NOS inhibitor was administered via i.c.v. The SUS tonus was reverted to its normal condition when the L-ARG drug was applied via i.c.v. In this specific situation, the NO can be considered an important neurotransmitter and/or neuromodulator of the functional activity of the lower urinary tract. It is reasonable to speculate that acetylcholine inhibition by L-SMTC via i.t. increases the inhibition of preganglionic parasympathetic nuclei (L_6_-S_1_ nuclei) and, indirectly, the inhibition of the intraneuronal segment, deactivating the pudendal nerve. Moreover, the results observed in this research suggest that the supraspinal neurons that express NOS can activate the midbrain periaqueductal gray and periventricular neurons that integrate the PMC (Barrington's nucleus).
